# Recent cryogenic electron microscopy structures of human A2M may not be representative of the native protein

**DOI:** 10.1073/pnas.2210218119

**Published:** 2022-08-16

**Authors:** Seandean Lykke Harwood, Gregers Rom Andersen, Jan J. Enghild

**Affiliations:** ^a^Department of Molecular Biology and Genetics, Aarhus University, 8000 Aarhus, Denmark

**Fig. 1.**
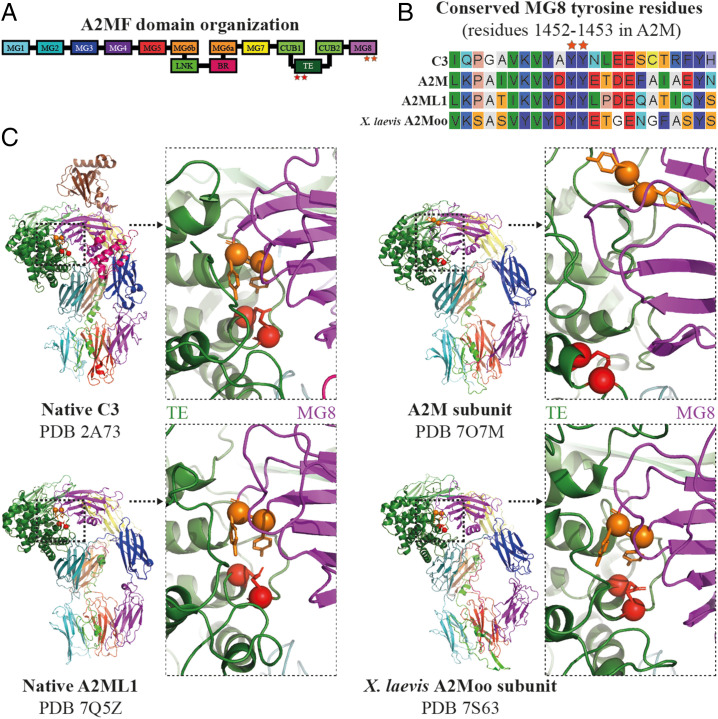
Comparison of the TE and MG8 domain interface in A2MF structures. (*A*) A schematic illustration of the domain organization of a typical A2MF protein. (*B*) Sequence alignment of human C3, A2M, and A2ML1, as well as A2Moo from the frog *X. laevis* in the vicinity of the conserved tyrosine residues in the MG8 domain. The tyrosine residues are indicated with orange stars and are highly conserved. (*C*) Cartoon representations of the structures of native C3 (PDB 2A73), an A2M subunit (PDB 7O7M), native A2ML1 (PDB 7Q5Z), and a frog A2Moo subunit (PDB 7S63). The TE cysteine and glutamine residues are shown in red and the conserved MG8 tyrosine residues are shown in orange, all with sphere C_α_ and stick side-chain representations. Domains are colored as indicated in *A*. Each structure is shown in its entirety and zoomed in toward the TE/MG8 domain interface. In C3, A2ML1, and A2Moo the TE is closely shielded by the MG8 tyrosines, whereas in the A2M subunit the TE is distant from the tyrosines.

Luque et al. have described multiple structures of human alpha-2-macroglobulin (A2M) determined from samples purified from frozen human plasma (1). The authors interpreted two of these structures (Protein Data Bank [PDB] entries 7O7M and 7O7L) as native A2M, i.e., representative of A2M in its original, physiological state before proteolysis and with an intact thiol ester (TE). The other structures are proposed to represent intermediate or final states occurring during A2M’s proteolysis-induced conformational change. However, there are significant differences between the two putatively native A2M structures and other determined structures of native proteins in the A2M family (A2MF), including complement factor C3 (2), A2M-like protein 1 (A2ML1) (3), and ovomacroglobulin (A2Moo) from the frog *Xenopus laevis* (4). In these proteins, the TE is protected in a hydrophobic environment formed by the TE and macroglobulin 8 (MG8) domains. In particular, two highly conserved MG8 tyrosine residues (Fig. 1 *A* and *B*) are arranged in a nearly identical fashion around the TE and restrict its access to water. This arrangement is not present in the 7O7M and 7O7L entries, where the MG8 tyrosines are positioned far from the TE, exposing it to solvent (Fig. 1*C*). Furthermore, the distance between the Cys972 S_γ_ and Gln975 C_δ_ atoms is 3.4 to 5.5 Å in these two entries, which is inconsistent with the expected ∼1.7-Å bond length of a TE. Considering these differences alongside the otherwise high degree of similarity in A2MF proteins’ structures and function, it is unlikely that the 7O7M and 7O7L structures are representative of A2M’s native structure. Possibly, they are conformational intermediates of A2M with hydrolyzed TEs; similar intermediates have been observed for complement C3 (5).

Approximately 75% of A2M molecules in the samples were not native according to the authors, and this percentage increases if the 7O7M and 7O7L structures are also nonnative. In contrast, the typical percentage of nonnative A2M in circulation is less than 1% (6), as nonnative A2M is rapidly cleared from circulation in the liver (7). Using well-established purification protocols that do not separate A2M’s conformational states, homogeneously native A2M (as determined by pore-limited native polyacrylamide gel electrophoresis and LRP1 binding) can be purified from fresh plasma (8). Therefore, it seems likely that the A2M samples used by Luque et al. (1) were damaged in some way. Freezing has been shown to alter the structure and functionality of A2M (9) and other A2MF proteins such as complement C3 (10). The A2M samples used by Luque et al. (1) were purified from frozen plasma, which may explain the sample heterogeneity, the low content of native A2M, and the probably nonnative 7O7M and 7O7L structures.
